# Supplementary material for: Study on the induction of exogenous plant hormones to enhance the weed suppression ability of allelopathic and non-allelopathic rice accessions
Source: PeerJ. 2025 Jul 15;13:e19700. doi: 10.7717/peerj.19700 (PMC12273701; doi:10.7717/peerj.19700)
Supplement: Supplemental Information 1 [file peerj-13-19700-s001.docx]

Table S1 Effect of 10 plant hormones on root growth of two rice cultivars

| plant hormones | root traits | rice cultivars | hormones concentration | | | |  |
| --- | --- | --- | --- | --- | --- | --- | --- |
|  |  |  |  |  |  |  |  |
|  |  |  | CK | 2 μmol/L | 10 μmol/L | 20 μmol/L |  |
| indole-3-acetic acid | root length (cm/plant) | PI | 382.10±21.62a | 355.57±4.65a | 301.13±21.17b | 293.60±1.42b |  |
|  |  | LE | 181.33±12.25b | 218.07±5.34a | 173.47±6.45b | 214.50±6.43a |  |
|  | root fresh height（g/plant） | PI | 0.653±0.002b | 0.705±0.007a | 0.689±0.007a | 0.649±0.006b |  |
|  |  | LE | 0.554±0.003b | 0.599±0.010a | 0.595±0.006a | 0.590±0.004a |  |
|  | root dry weight（g/plant） | PI | 0.067±0.002a | 0.068±0.001a | 0.067±0.001a | 0.061±0.001b |  |
|  |  | LE | 0.062±0.001b | 0.071±0.002a | 0.069±0.001a | 0.070±0.001a |  |
|  |  |  | CK | 0.1 mg/L | 0.5 mg/L | 1 mg/L |  |
| indole-3-butyric acid | root length (cm/plant) | PI | 510.55±14.19a | 398.05±15.60b | 359.15±25.66b | 333.03±28.91b |  |
|  |  | LE | 273.93±9.59a | 266.96±21.90a | 230.98±26.05a | 230.55±18.72a |  |
|  | root fresh height（g/plant） | PI | 1.062±0.035a | 0.818±0.032b | 0.775±0.016b | 0.773±0.041b |  |
|  |  | LE | 0.425±0.031a | 0.468±0.049a | 0.455±0.041a | 0.496±0.066a |  |
|  | root dry weight（g/plant） | PI | 0.093±0.003a | 0.082±0.004a | 0.079±0.002a | 0.078±0.008a |  |
|  |  | LE | 0.040±0.003a | 0.044±0.005a | 0.045±0.004a | 0.050±0.006a |  |
|  |  |  | CK | 0.1 mg/L | 0.5 mg/L | 1 mg/L |  |
| 1-naphthalene acetic acid | root length (cm/plant) | PI | 240.35±20.18a | 247.92±17.64a | 240.92±11.06a | 245.55±14.75a |  |
|  |  | LE | 380.07±20.39a | 257.65±9.71b | 226.28±4.16b | 237.33±16.02b |  |
|  | root fresh height（g/plant） | PI | 0.731±0.029b | 0.836±0.157a | 0.730±0.028b | 0.648±0.081b |  |
|  |  | LE | 0.759±0.055a | 0.684±0.028ab | 0.556±0.028c | 0.556±0.016bc |  |
|  | root dry weight（g/plant） | PI | 0.062±0.007c | 0.076±0.003ab | 0.084±0.001a | 0.069±0.004bc |  |
|  |  | LE | 0.063±0.003ab | 0.070±0.003a | 0.057±0.002b | 0.060±0.003ab |  |

Note: The lowercase letters represent significant difference at *p*<0.05 level.

Table S1 (Continued)

| plant hormones | root traits | rice cultivars | hormones concentration | | | |
| --- | --- | --- | --- | --- | --- | --- |
|  |  |  |  |  |  |  |
|  |  |  | CK | 0.34 μmol/L | 1.36 μmol/L | 3.40 μmol/L |
| ethephon | root length (cm/plant) | PI | 360.77±4.36b | 387.67±6.99a | 375.90±10.11ab | 323.00±6.41c |
|  |  | LE | 263.23±3.78b | 340.67±18.52a | 306.73±27.85ab | 293.7±10.0ab |
|  | root fresh height（g/plant） | PI | 0.689±0.039b | 0.751±0.057ab | 0.886±0.008a | 0.812±0.052ab |
|  |  | LE | 0.524±0.014c | 0.746±0.027a | 0.594±0.022b | 0.626±0.020b |
|  | root dry weight（g/plant） | PI | 0.055±0.005b | 0.059±0.003b | 0.077±0.001a | 0.064±0.005b |
|  |  | LE | 0.042±0.002b | 0.052±0.004a | 0.049±0.001ab | 0.050±0.002a |
|  |  |  | CK | 1 μmol/L | 5 μmol/L | 10 μmol/L |
| gibberellins | root length (cm/plant) | PI | 370.30±28.16a | 282.87±18.83b | 323.55±9.41ab | 320.38±15.73ab |
|  |  | LE | 258.90±10.00a | 222.00±10.77ab | 214.12±11.79b | 199.89±19.35b |
|  | root fresh height（g/plant） | PI | 0.655±0.062a | 0.570±0.034a | 0.584±0.039a | 0.598±0.051a |
|  |  | LE | 0.551±0.043a | 0.391±0.026b | 0.396±0.032b | 0.388±0.033b |
|  | root dry weight（g/plant） | PI | 0.056±0.005a | 0.049±0.003a | 0.049±0.003a | 0.049±0.004a |
|  |  | LE | 0.043±0.003a | 0.031±0.002b | 0.032±0.002b | 0.033±0.003b |
|  |  |  | CK | 1 μmol/L | 5 μmol/L | 10μmol/L |
| abscisic acid | root length (cm/plant) | PI | 213.60±7.32b | 239.33±7.68ab | 246.63±18.31a | 217.87±2.03ab |
|  |  | LE | 205.60±2.38c | 229.93±9.47b | 263.23±5.27a | 249.80±5.85a |
|  | root fresh height（g/plant） | PI | 0.316±0.027bc | 0.386±0.024ab | 0.403±0.022a | 0.285±0.016c |
|  |  | LE | 0.353±0.020b | 0.383±0.018b | 0.464±0.016a | 0.363±0.024b |
|  | root dry weight（g/plant） | PI | 0.033±0.004b | 0.043±0.004ab | 0.045±0.003a | 0.036±0.001ab |
|  |  | LE | 0.034±0.001b | 0.037±0.003b | 0.045±0.002a | 0.038±0.002b |

Note: The lowercase letters represent significant difference at *p*<0.05 level.

Table S1 (Continued)

| name | root traits | rice | concentration | | | |
| --- | --- | --- | --- | --- | --- | --- |
|  |  |  |  |  |  |  |
|  |  |  | CK | 0.01 nmol/L | 0.05 nmol/L | 0.1 nmol/L |
| brassinolide | root length (cm/plant) | PI | 332.57±7.66b | 551.07±3.36a | 506.93±30.49a | 527.03±16.70a |
|  |  | LE | 194.83±4.05b | 246.73±6.90a | 252.23±14.74a | 253.47±7.74a |
|  | root fresh height (g/plant) | PI | 0.847±0.017c | 1.158±0.029a | 0.945±0.012b | 0.999±0.042b |
|  |  | LE | 0.761±0.009c | 1.058±0.009a | 0.970±0.049ab | 0.939±0.032b |
|  | root dry weight (g/plant) | PI | 0.079±0.002c | 0.100±0.002ab | 0.101±0.001a | 0.095±0.002b |
|  |  | LE | 0.063±0.001c | 0.090±0.001a | 0.078±0.003b | 0.074±0.002b |
|  |  |  | CK | 0.1 nmol/L | 0.5 nmol/L | 1 nmol/L |
| 24-epibrassinolide | root length (cm/plant) | PI | 376.47±9.00b | 483.87±8.12a | 497.07±47.19a | 510.70±31.53a |
|  |  | LE | 205.73±12.49b | 291.70±34.56a | 283.10±13.48a | 309.70±5.36a |
|  | root fresh height (g/plant) | PI | 0.999±0.022c | 1.228±0.031ab | 1.229±0.016a | 1.151±0.023b |
|  |  | LE | 0.791±0.009b | 1.025±0.043a | 1.021±0.022a | 0.967±0.030a |
|  | root dry weight (g/plant) | PI | 0.099±0.003c | 0.112±0.001ab | 0.120±0.005a | 0.109±0.002bc |
|  |  | LE | 0.065±0.002c | 0.080±0.001a | 0.082±0.001a | 0.073±0.003b |
|  |  |  | CK | 0.1 μmol/L | 1 μmol/L | 10 μmol/L |
| jasmonic acid | root length (cm/plant) | PI | 271.17±0.84b | 302.20±5.80a | 276.97±3.87ab | 244.23±13.79c |
|  |  | LE | 177.00±9.49b | 202.27±3.70a | 175.13±5.66b | 159.10±1.29b |
|  | root fresh height (g/plant) | PI | 0.425±0.004ab | 0.443±0.012a | 0.402±0.006b | 0.357±0.001c |
|  |  | LE | 0.431±0.007b | 0.522±0.015a | 0.490±0.014a | 0.374±0.012c |
|  | root dry weight (g/plant) | PI | 0.041±0.001ab | 0.043±0.001a | 0.041±0.000ab | 0.040±0.000b |
|  |  | LE | 0.043±0.001b | 0.050±0.002a | 0.047±0.000a | 0.042±0.001b |
|  |  |  | CK | 0.1 μmol/L | 1 μmol/L | 10 μmol/L |
| methyl jasmonate | root length (cm/plant) | PI | 284.63±2.76b | 307.83±8.51a | 220.80±8.91c | 181.40±2.44d |
|  |  | LE | 238.90±6.32b | 269.73±14.48a | 212.43±5.42b | 170.13±1.86c |
|  | root fresh height (g/plant) | PI | 0.507±0.007b | 0.535±0.005a | 0.390±0.011c | 0.330±0.004d |
|  |  | LE | 0.655±0.011b | 0.732±0.007a | 0.516±0.018c | 0.429±0.001d |
|  | root dry weight (g/plant) | PI | 0.049±0.001b | 0.061±0.002a | 0.050±0.001b | 0.046±0.002b |
|  |  | LE | 0.064±0.001b | 0.078±0.001a | 0.059±0.001c | 0.059±0.001c |

Note: The lowercase letters represent significant difference at *p*<0.05 level.

Table S2 Primers for quantitative real time-PCR

| Gene name | Forward primer (5’→ 3’) | Reverse primer (5’→ 3’) |
| --- | --- | --- |
| phenylalanine ammonia-lyase (*PAL*) | AAGGTGTTCCTCGGCATCAG | AGCGGTTCGTTTTCTTTTAT |
| cinnamate-4-hydroxylase (*C4H*) | AACCACCCGAGCATCCAG | AACCGCAGCGTCTCCTTC |
| ferulic acid -5-hydroxylases (*F5H*) | TACTCCCAGGACCCCAACG | GCGACACCAGCACGAACC |
| caffeic acid 3-O-methyltransferase (*COMT*) | GACAGCACCAACAAGA | TGAAGACACCCACCTC |
| 3-Hydroxy-3-methylglutar yl-CoA reductase (*HMGR*) | CTCAGGTAACTTTTGCTC | CAGTGGCTATGAAGATTG |
| mevalonate kinase (*MK*) | CAGTCGGCGGCATCAG | CACCAGCAACCAGAGC |
| monoterpenes synthase (*MTS*) | GGATGAACTTGGGATTGG | TCTGGAGAAGGAAGCACC |
| sesquiterpene synthase (*STS*) | CGATGATAACAATAACGG | GTGCCTTGCTTCAACTCT |
| phytoene synthase (*PS*) | ATGGAGATGTTTTATGAGGGA | CGTCAAGGATTTGTCGGT |
| squalene synthase (*SQS*) | GTTTCGCCTTGTCTCCAC | CAACAGTTTCAACCTCCTT |


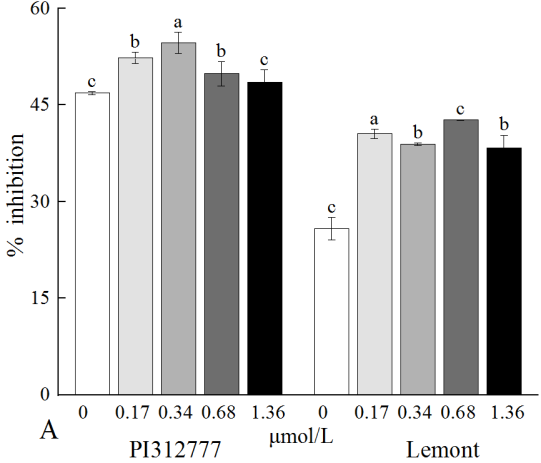

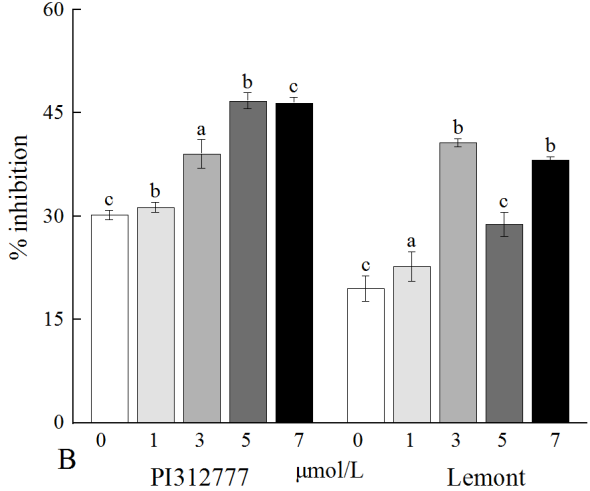


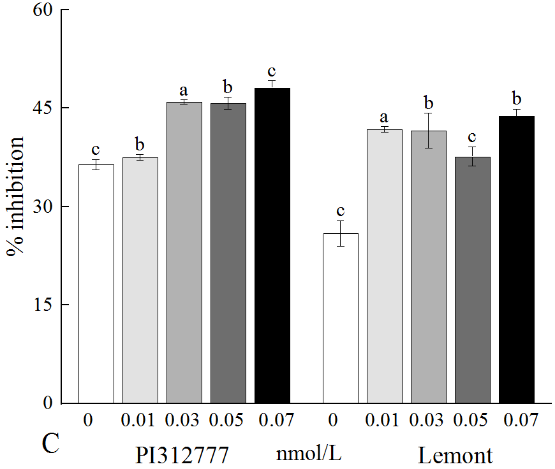

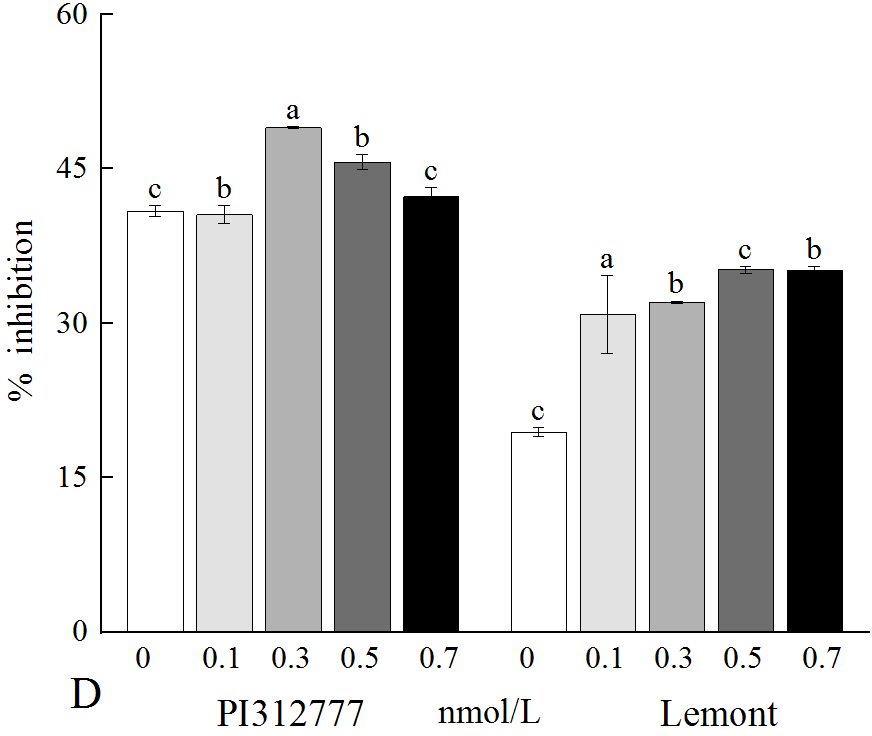


Figure S1 The inhibition rate (%) of rice leaf extracts treated with ETH(A), ABA(B), BR(C), EBL (D) after 7 days. The lowercase letters indicate significant differences at the *p*<0.05 level between induction concentrations of the same hormone in the same rice.


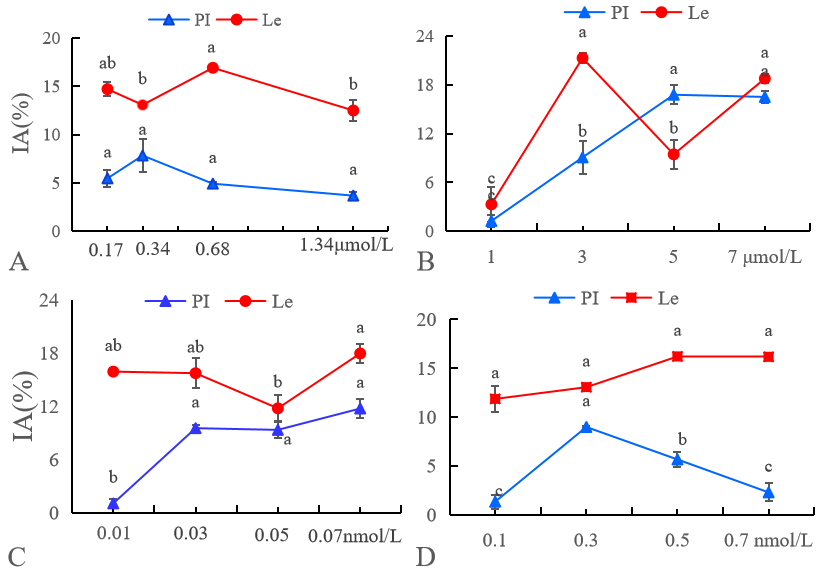


Figure S2 Effects of ETH(A), ABA(B), BR(C), EBL (D) on rice induced allelopathy (IA) at different concentrations. Lowercase letters indicate significant differences at the *p*<0.05 level between induction concentrations of the same hormone in the same rice.

Table S3 Contents of terpenoids in the leaf-extracts of allelopathic rice PI without induction

| Species of terpenoids | Compound Name | formula | Concentration（μg/g plant) |
| --- | --- | --- | --- |
| oxygenated monoterpene | 2-Decenal, (E)- | C_10_H_18_O | 1.02 |
|  | 7-Oxabicyclo[2.2.1]heptane, 1-methyl-4-(1-methylethyl)- | C_10_H_18_O | 0.14 |
|  | Levomenthol | C_10_H_20_O | 0.35 |
|  | 10-Undecenal | C_11_H_20_O | 0.14 |
|  | Cyclododecanecarboxylic acid | C_13_H_24_O_2_ | 0.44 |
|  | Citronellyl butyrate | C_14_H_26_O_2_ | 2.27 |
|  | Citronellyl isobutyrate | C_14_H_26_O_2_ | 0.31 |
|  | Tetradecanal | C_14_H_28_O | 0.60 |
| non-oxygenated  monoterpene | Naphthalene, 1-methyl- | C_11_H_10_ | 0.15 |
|  | 1,4-Methanonaphthalene, 1,4-dihydro- | C_11_H_10_ | 0.38 |
|  | Naphthalene, 1,6,7-trimethyl- | C_13_H_14_ | 0.13 |
|  | Dodecane, 2-methyl- | C_13_H_28_ | 0.14 |
|  | Dodecane, 4,6-dimethyl- | C_14_H_30_ | 0.19 |
| oxygenated sesquiterpene | Hexadecanal | C_16_H_32_O | 0.77 |
|  | Dibutyl phthalate | C_16_H_22_O_4_ | 0.69 |
|  | 2-Pentadecanone, 6,10,14-trimethyl- | C_18_H_36_O | 0.64 |
|  | Octadecanal | C_18_H_36_O | 4.20 |
|  | Linolenic acid, methyl ester | C_19_H_32_O_2_ | 0.26 |
|  | Methoprene | C_19_H_34_O_3_ | 0.85 |
| non-oxygenated  sesquiterpene | Dodecane, 2,6,11-trimethyl- | C_15_H_32_ | 3.75 |
|  | Dodecane, 2,6,10-trimethyl- | C_15_H_32_ | 0.17 |
|  | Heptadecane, 8-methyl- | C_18_H_38_ | 0.70 |
|  | cis-2-Methyl-7-octadecene | C_19_H_38_ | 0.94 |
| oxygenated diterpenes | Phytol | C_20_H_40_O | 2.34 |
|  | Sclareol | C_20_H_36_O_2_ | 1.37 |
|  | Methyl 11,14,17-icosatrienoate | C_21_H_36_O_2_ | 0.38 |
|  | Batilol | C_21_H_44_O_3_ | 0.18 |
|  | Strophanthidin | C_23_H_32_O_6_ | 0.13 |
|  | Lanosterol | C_30_H_50_O | 1.32 |
| non-oxygenated  diterpenes | Hexadecane, 2,6,10,14-tetramethyl- | C_20_H_42_ | 0.80 |
|  | Octadecane, 9-ethyl-9-heptyl- | C_27_H_56_ | 0.35 |
| total |  |  | 26.09 |

Table S4 Contents of terpenoids in the leaf-extracts of allelopathic rice PI after ABA induction

| Species of terpenoids | Compound Name | formula | Concentration（μg/g plant) |
| --- | --- | --- | --- |
| oxygenated monoterpene | 2-Decenal, (E)- | C_10_H_18_O | 0.86 |
|  | 1-Butanone, 1-cyclohexyl- | C_10_H_18_O | 0.55 |
|  | Citronellal | C_10_H_18_O | 1.32 |
|  | Heptanedioic acid, 3-methyl-, dimethyl ester | C_10_H_18_O_4_ | 1.07 |
|  | Cyclohexanol, 5-methyl-2-(1-methylethyl)- | C_10_H_20_O | 6.17 |
|  | Levomenthol | C_10_H_20_O | 1.74 |
|  | 1,2-Epoxy-5,9-cyclododecadiene | C_12_H_18_O | 0.70 |
|  | 6-Octen-1-ol, 3,7-dimethyl-, acetate | C_12_H_22_O_2_ | 1.13 |
|  | 2-Butanone, 4-(2,6,6-trimethyl-1-cyclohexen-1-yl)- | C_13_H_22_O | 2.15 |
|  | Tetradecanal | C_14_H_28_O | 0.65 |
|  | 2-Propen-1-one, 1-(2-hydroxyphenyl)-3-(3-hydroxyphenyl)- | C_15_H_12_O_3_ | 0.54 |
| non-oxygenated  monoterpene | Naphthalene, 2-methyl- | C_11_H_10_ | 0.79 |
|  | Dodecane, 2,6,10-trimethyl- | C_15_H_32_ | 0.79 |
| oxygenated sesquiterpene | Palustrol | C_15_H_26_O | 1.31 |
|  | Patchouli alcohol | C_15_H_26_O | 1.42 |
|  | Cedrol | C_15_H_26_O | 0.93 |
|  | Glucitol, 6-O-nonyl- | C_15_H_32_O_6_ | 0.57 |
|  | cis-11-Hexadecenal | C_16_H_30_O | 6.36 |
|  | Equilenin | C_18_H_18_O_2_ | 1.25 |
|  | 2-Pentadecanone, 6,10,14-trimethyl- | C_18_H_36_O | 0.83 |
|  | 9-Octadecen-1-ol, (Z)- | C_18_H_36_O | 8.73 |
|  | Hexadecanoic acid, 2-hydroxyethyl ester | C_18_H_36_O_3_ | 0.72 |
|  | 9,15-Octadecadienoic acid, methyl ester, | C_19_H_34_O_2_ | 2.67 |
|  | Methoprene | C_19_H_34_O_3_ | 3.14 |
| non-oxygenated  sesquiterpene | (-)-α-gurjunene | C_15_H_24_ | 1.09 |
|  | Sparteine | C_15_H_26_N_2_ | 0.57 |
|  | cis-2-Methyl-7-octadecene | C_19_H_38_ | 0.57 |
| oxygenated diterpenes | Linolenic acid, ethyl ester | C_20_H_34_O_2_ | 1.04 |
|  | Phytol | C_20_H_40_O | 1.55 |
|  | 11,14,17-Eicosatrienoic acid, methyl ester | C_21_H_36_O_2_ | 0.57 |
| non-oxygenated  diterpenes | Hexadecane, 2,6,10,14-tetramethyl- | C_20_H_42_ | 0.72 |
| total |  |  | 49.72 |

Table S5 Contents of terpenoids in the leaf-extracts of allelopathic rice PI after EBL induction

| Species of terpenoids | Compound Name | formula | Concentration（μg/g plant) |
| --- | --- | --- | --- |
| oxygenated monoterpene | Dextro-camphoric acid | C_10_H_16_O_4_ | 1.07 |
|  | 2-Decenal, (E)- | C_10_H_18_O | 1.06 |
|  | Isopulegol | C_10_H_18_O | 0.68 |
|  | Levomenthol | C_10_H_20_O | 1.45 |
|  | Citronellol | C_10_H_20_O | 0.58 |
|  | 10-Undecenal | C_11_H_20_O | 1.17 |
|  | trans-2-undecenoic acid | C_11_H_20_O_2_ | 0.63 |
|  | 1,2-Epoxy-5,9-cyclododecadiene | C_12_H_18_O | 0.79 |
|  | Tetradecanal | C_14_H_28_O | 1.55 |
| non-oxygenated  monoterpene | Dodecane, 2,6,11-trimethyl- | C_15_H_32_ | 0.55 |
|  | Dodecane, 2,6,10-trimethyl- | C_15_H_32_ | 0.55 |
| oxygenated sesquiterpene | Caryophyllene oxide | C_15_H_24_O | 0.60 |
|  | Patchouli alcohol | C_15_H_26_O | 1.50 |
|  | Isolongifolol | C_15_H_26_O | 1.04 |
|  | Cedrol | C_15_H_26_O | 2.85 |
|  | Citronellyl tiglate | C_15_H_26_O_2_ | 0.56 |
|  | Glucitol, 6-O-nonyl- | C_15_H_32_O_6_ | 1.79 |
|  | Musk ambrette | C_16_H_28_O_2_ | 0.60 |
|  | 2-Hexyl-1-decanol | C_16_H_34_O | 0.49 |
|  | 9-Octadecen-1-ol, (Z)- | C_18_H_36_O | 2.54 |
|  | Octadecanal | C_18_H_36_O | 0.80 |
|  | inolenic acid, methyl ester | C_19_H_32_O_2_ | 0.69 |
|  | Methoprene | C_19_H_34_O_3_ | 1.06 |
|  | Disparlure | C_19_H_38_O | 0.70 |
| non-oxygenated  sesquiterpene | 1-Octadecyne | C_18_H_34_ | 0.51 |
|  | Heptadecane, 8-methyl- | C_18_H_38_ | 0.63 |
| oxygenated diterpenes | Hydroxydehydrostevic acid | C_20_H_30_O_3_ | 0.63 |
|  | Phytol | C_20_H_40_O | 4.19 |
|  | Methyl 11,14,17-icosatrienoate | C_21_H_36_O_2_ | 0.45 |
|  | Ascorbyl Palmitate | C_22_H_38_O7 | 0.55 |
|  | 5-Cholenic acid-3.beta.-ol | C_24_H_38_O_3_ | 0.44 |
|  | Stigmasterol | C_29_H_48_O | 1.26 |
| non-oxygenated  diterpenes | 1-Eicosene | C_20_H_40_ | 7.37 |
|  | Hexadecane, 2,6,10,14-tetramethyl- | C_20_H_42_ | 1.02 |
|  | Cholest-4-ene | C_27_H_46_ | 0.90 |
| total |  |  | 43.25 |

Table S6 Contents of terpenoids in the leaf-extracts of non-allelopathic rice LE without induction

| Species of terpenoids | Compound Name | formula | Concentration（μg/g plant) |
| --- | --- | --- | --- |
| oxygenated monoterpene | Isopulegol | C_10_H_18_O | 0.94 |
|  | Decanal | C_10_H_20_O | 0.90 |
|  | Citronellol | C_10_H_20_O | 2.04 |
|  | Undecanal | C_11_H_22_O | 0.34 |
|  | trans-2-Undecen-1-ol | C_11_H_22_O | 1.20 |
|  | 1,2-Epoxy-5,9-cyclododecadiene | C_12_H_18_O | 2.31 |
|  | Tetradecanoic acid | C_14_H_28_O_2_ | 0.67 |
| non-oxygenated  monoterpene | Naphthalene, 2-methyl- | C_11_H_10_ | 0.29 |
| oxygenated sesquiterpene | Caryophyllene oxide | C_15_H_24_O | 0.30 |
|  | Cedrol | C_15_H_26_O | 2.85 |
|  | Geranyl isovalerate | C_15_H_26_O_2_ | 0.95 |
|  | Glucitol, 6-O-nonyl- | C_15_H_32_O_6_ | 0.55 |
|  | Hexadecanal | C_16_H_32_O | 1.43 |
|  | 1-Decanol, 2-hexyl- | C_16_H_34_O | 0.60 |
| non-oxygenated  sesquiterpene | Heptadecane, 8-methyl- | C_18_H_38_ | 0.80 |
| oxygenated diterpenes | Sclareol | C_20_H_36_O_2_ | 1.04 |
|  | Phytol | C_20_H_40_O | 0.72 |
|  | Methyl 11,14,17-icosatrienoate | C_21_H_36_O_2_ | 0.30 |
|  | Batilol | C_21_H_44_O_3_ | 1.78 |
|  | Behenic alcohol | C_22_H_46_O | 0.34 |
| non-oxygenated  diterpenes | Hexadecane, 2,6,10,14-tetramethyl- | C_20_H_42_ | 1.03 |
| total |  |  | 21.39 |

Table S7 Contents of terpenoids in the leaf-extracts of non-allelopathic rice LE after ABA induction

| Species of terpenoids | Compound Name | formula | Concentration（μg/g plant) |
| --- | --- | --- | --- |
| oxygenated monoterpene | 3-Thujanone | C_10_H_16_O | 0.71 |
|  | Dextro-camphoric acid | C_10_H_16_O_4_ | 2.72 |
|  | Decanal | C_10_H_20_O | 0.75 |
|  | Menthol | C_10_H_20_O | 0.32 |
|  | Levomenthol | C_10_H_20_O | 0.43 |
|  | Cyclohexanepropanoic acid, 2-propenyl ester | C_12_H_20_O_2_ | 0.46 |
|  | trans-.beta.-Ionone | C_13_H_20_O | 0.68 |
|  | Citronellyl isobutyrate | C_14_H_26_O_2_ | 0.83 |
|  | Tetradecanal | C_14_H_28_O | 3.36 |
| non-oxygenated  monoterpene | Dodecane, 4,6-dimethyl- | C_14_H_30_ | 0.76 |
| oxygenated sesquiterpene | Costunolide | C_15_H_20_O_2_ | 1.21 |
|  | (-)-Spathulenol | C_15_H_24_O | 0.47 |
|  | Bisabolol | C_15_H_26_O | 0.35 |
|  | Patchouli alcohol | C_15_H_26_O | 0.57 |
|  | Cedrol | C_15_H_26_O | 2.21 |
|  | Dihydro-.beta.-agarofuran | C_15_H_26_O | 0.29 |
|  | 1-Dodecanol, 3,7,11-trimethyl- | C_15_H_32_O | 0.33 |
|  | 9-Tetradecen-1-ol, acetate, (Z)- | C_16_H_30_O_2_ | 0.66 |
|  | Hexadecanal | C_16_H_32_O | 0.53 |
|  | 1-Decanol, 2-hexyl- | C_16_H_34_O | 0.73 |
|  | Isopropyl myristate | C_17_H_34_O_2_ | 0.60 |
|  | 9-Octadecen-1-ol, (Z)- | C_18_H_36_O | 1.63 |
|  | Oxirane, hexadecyl- | C_18_H_36_O | 0.57 |
|  | Octadecanal | C_18_H_36_O | 0.56 |
| non-oxygenated  sesquiterpene | Dodecane, 2,6,10-trimethyl- | C_15_H_32_ | 1.19 |
|  | 1-Nonadecene | C_19_H_38_ | 0.57 |
| oxygenated diterpenes | cis-8,11,14-Eicosatrienoic Acid | C_20_H_34_O_2_ | 1.66 |
|  | Phytol | C_20_H_40_O | 6.36 |
|  | Methyl 11,14,17-icosatrienoate | C_21_H_36_O_2_ | 0.51 |
|  | Batilol | C_21_H_44_O_3_ | 0.57 |
| non-oxygenated  diterpenes | Hexadecane, 2,6,10,14-tetramethyl- | C_20_H_42_ | 0.95 |
| total |  |  | 33.52 |

Table S8 Contents of terpenoids in the leaf-extracts of non-allelopathic rice LE after EBL induction

| Species of terpenoids | Compound Name | formula | Concentration（μg/g plant) |
| --- | --- | --- | --- |
| oxygenated monoterpene | Isopulegol | C_10_H_18_O | 0.31 |
|  | Decanal | C_10_H_20_O | 1.51 |
|  | trans-2-undecenoic acid | C_11_H_20_O_2_ | 0.37 |
|  | Undecanal | C_11_H_22_O | 0.14 |
|  | 1,2-Epoxy-5,9-cyclododecadiene | C_12_H_18_O | 0.14 |
|  | trans-Traumatic acid | C_12_H_20_O_4_ | 1.80 |
|  | 2-Butanone, 4-(2,2,6-trimethylcyclohexyl)- | C_13_H_24_O | 0.61 |
| non-oxygenated  monoterpene | Dodecane, 2-methyl- | C_13_H_28_ | 0.18 |
|  | Dodecane, 2,6,11-trimethyl- | C_15_H_32_ | 0.13 |
|  | Dodecane, 2,6,10-trimethyl- | C_15_H_32_ | 0.63 |
| oxygenated sesquiterpene | Caryophyllene oxide | C_15_H_24_O | 0.27 |
|  | Isolongifolol | C_15_H_26_O | 0.39 |
|  | Patchouli alcohol | C_15_H_26_O | 3.25 |
|  | Geranyl isovalerate | C_15_H_26_O_2_ | 0.46 |
|  | Sclareolide | C_16_H_26_O_2_ | 0.76 |
|  | 7-Hexadecenal, (Z)- | C_16_H_30_O | 2.97 |
|  | Hexadecanal | C_16_H_32_O | 0.82 |
|  | Isopropyl myristate | C_17_H_34_O_2_ | 0.41 |
|  | Oxirane, hexadecyl- | C_18_H_36_O | 0.15 |
|  | Methoprene | C_19_H_34_O_3_ | 6.77 |
| non-oxygenated  sesquiterpene | Isolongifolene | C_15_H_24_ | 0.25 |
|  | Heptadecane, 8-methyl- | C_18_H_38_ | 0.36 |
| oxygenated diterpenes | Sclareol | C_20_H_36_O_2_ | 0.15 |
|  | Phytol | C_20_H_40_O | 0.19 |
|  | 11,14-Eicosadienoic acid, methyl ester | C_21_H_38_O_2_ | 0.32 |
|  | Batilol | C_21_H_44_O_3_ | 0.21 |
|  | Behenic alcohol | C_22_H_46_O | 1.89 |
|  | Strophanthidin | C_23_H_32_O_6_ | 0.40 |
|  | Tetracosanoic acid | C_24_H_48_O_2_ | 0.27 |
| non-oxygenated  diterpenes | Hexadecane, 2,6,10,14-tetramethyl- | C_20_H_42_ | 0.17 |
|  | 9-Tricosene, (Z)- | C_23_H_46_ | 2.77 |
| total |  |  | 29.04 |
